# Common, Less Common, and Unexpected Complications after Bariatric Surgery: A Pictorial Essay

**DOI:** 10.3390/diagnostics12112637

**Published:** 2022-10-31

**Authors:** Francesca Iacobellis, Giuseppina Dell’Aversano Orabona, Antonio Brillantino, Marco Di Serafino, Alessandro Rengo, Paola Crivelli, Luigia Romano, Mariano Scaglione

**Affiliations:** 1 Department of General and Emergency Radiology, “A. Cardarelli” Hospital, Via A. Cardarelli 9, 80131 Naples, Italy; 2 Department of Emergency Surgery, “A. Cardarelli” Hospital, Via A. Cardarelli 9, 80131 Naples, Italy; 3 Department of Radiology, Pineta Grande Hospital, Via Domitiana KM 30, 81030 Castel Volturno, Italy; 4 Department of Surgery, Medicine and Pharmacy, University of Sassari, Via Roma 151, 07100 Sassari, Italy; 5 Department of Radiology, The James Cook University Hospital, Middlesbrough TS4 3BW, UK; 6 School of Health and Life Sciences, Teesside University, Middlesbrough TS1 3BX, UK

**Keywords:** bariatric surgery, bariatric surgery complications, obesity, sleeve gastrectomy, gastric banding, gastric bypass, imaging, computed tomography, leak, emergency

## Abstract

Bariatric surgery has demonstrated a higher rate of success than other nonsurgical treatments in selected patients with obesity; however, like all medical procedures, postoperative complications may occur, ranging between 2 and 10% and, although rare, they can be life threatening. Complications may be unspecific (any surgery-related complications) or specific (linked to the specific surgical procedure) and can be distinguished as common, less common, and unexpected. According to the onset, they may be acute, when occurring in the first 30 days after surgery, or chronic, with a presentation after 30 days from the procedure. The aim of this pictorial essay is to review the radiological aspects of surgical techniques usually performed and the possible complications, in order to make radiologists more confident with the postsurgical anatomy and with the normal and abnormal imaging findings.

## 1. Introduction

The World Health Organization’s (WHO) definition of excessive weight and obesity is “abnormal or excessive accumulation of fat with associated health risk”. A normal body mass index (BMI) is considered as 18.5–24.9 kg/m^2^, but being overweight carries a BMI ≥25, obesity has a BMI of ≥30 kg/m^2^, and severe obesity has a BMI of ≥40 kg/m^2^. The worldwide prevalence of these entities has doubled in the last 40 years, and a third of world population is today considered as overweight or obese, with an important increase of healthcare costs in relation to the increased risk of these patients to develop multiple comorbidities [1].

According to the American Society for Metabolic and Bariatric Surgery (ASMBS), patients are eligible for bariatric surgery when a BMI is ≥40 kg/m^2^, or more than 100 pounds overweight, or a BMI ≥35 kg/m^2^ and at least one or more obesity-related co-morbidities, such as type II diabetes, hypertension, sleep apnea and other respiratory disorders, non-alcoholic fatty liver disease, osteoarthritis, lipid abnormalities, gastrointestinal disorders, or heart disease, and an inability to achieve healthy and long-term weight loss after prior weight loss efforts [2,3].

Bariatric surgery has demonstrated a higher rate of success than other nonsurgical treatments in selected patients [4,5]; however, like all medical procedures, postoperative complications may occur, ranging between 2 and 10% and, although rare, they can be life threatening [4,5,6].

Complications may be unspecific (any surgery-related complications) or specific (linked to the specific surgical procedure) [7] and can be distinguished as common, less common, and unexpected.

According to the onset, they may be acute, when occurring in the first 30 days after surgery, or late, with a presentation after 30 days from the procedure [8].

The aim of this pictorial essay is to review the radiological aspects of surgical techniques usually performed and the possible complications, to make radiologists more confident with the postsurgical anatomy and with the normal and abnormal imaging findings.

## 2. Bariatric Surgical Techniques

To adequately assess complications of bariatric surgery one needs to know the surgical techniques usually performed which can be basically distinguished as restrictive and combined restricted and malabsorptive procedures; however, much more complex mechanisms are involved in the weight loss and related metabolic benefits [9]. 

### 2.1. Restrictive Procedures

Restrictive procedures include gastric balloon (GB), adjustable gastric banding (AGB), sleeve gastrectomy (SG), and clip gastroplasty (CP).

**Figure 1:**
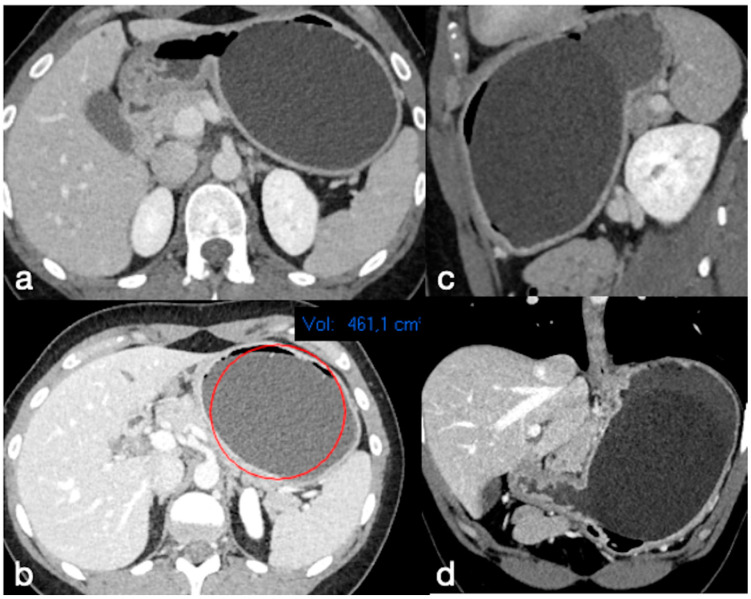
A well-placed gastric balloon in the gastric corpus and antrum seen at CT in axial (**a**,**b**), sagittal (**c**), and coronal (**d**) views, with an estimated volume of 461 cc (**b**).

GB is an expandable (400 mL or greater) silicone balloon endoscopically placed in the stomach, in order to reduce food intake, for a period of six months or less (Figure 1) [10]. The weight-loss mechanism of intragastric balloon therapy is restrictive; indeed, any balloon with a volume of 400 mL or greater can induce satiety, and so the patient decreases the amount of food intake (Figure 1) [11].

**Figure 2:**
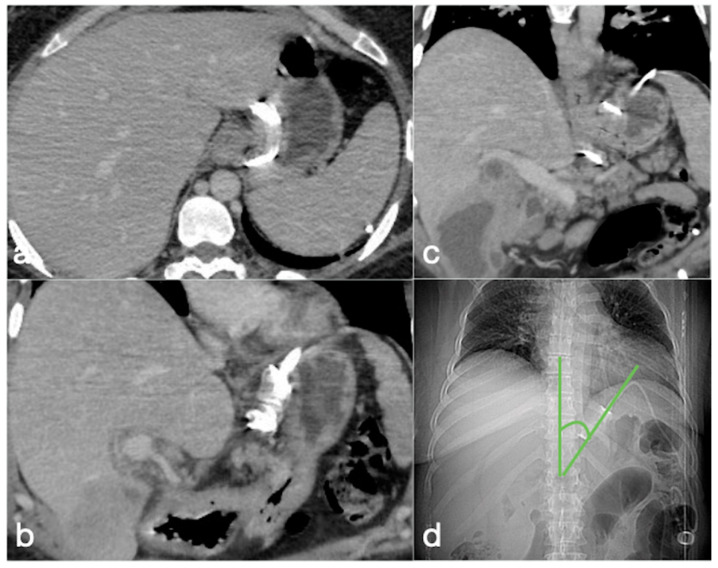
Correct positioning of the gastric banding seen in the CT axial (**a**) and coronal (**b**,**c**) views. (**d**) The CT localizer at the phi angle. This is the angle that needs to be reviewed to confirm that the gastric band is normally positioned. This is formed by the profile of the gastric band and the vertical axis of the spine on frontal view. The normal range is between 4° and 58°. In the shown case, it is at 40°.

AGB consists of a silicone band with an inflatable balloon cuff placed around the proximal stomach, approximately 2 cm distal to the gastro-esophageal junction, creating a small gastric pouch in communication with the remnant stomach (Figure 2). The anterior gastric wall can be sutured over the band to the gastric pouch to decrease the chances of band slippage. The band is then tubing-connected to a port placed subcutaneously in the abdomen. Through percutaneous aspiration or injection of saline or radio-opaque contrast into the port, the size of the balloon inside the band can be decreased or increased, thereby adjusting the stomal width [12].

**Figure 3:**
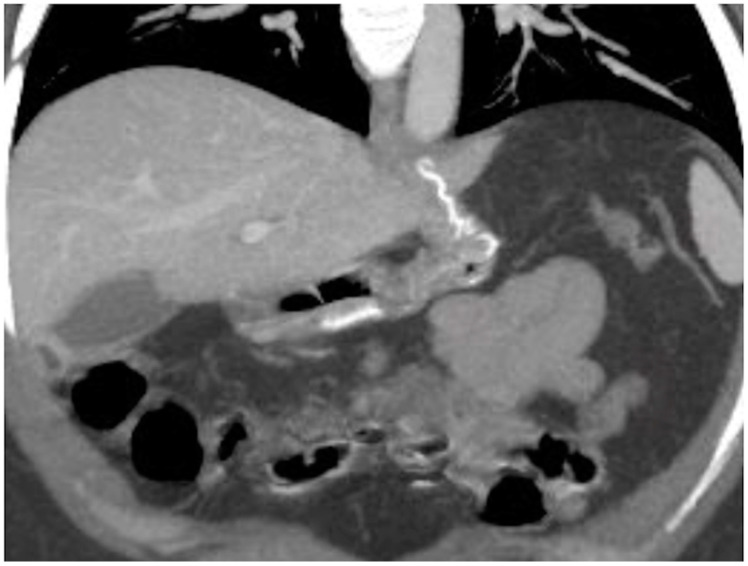
Surgical suture of sleeve gastrectomy seen on CT coronal oblique CT view.

In the SG procedure, the stomach is divided according to its longitudinal axis with resection of the fundus and great curvature side and leaving a thin gastric pouch of approximately 100 mL along the lesser curve (Figure 3). The new smaller stomach or “sleeve,” forces the patient to eat smaller portions, and consume fewer calories. In addition, there is a hormonal benefit related with increased production of hormones that positively influences satiety [4,9,13,14].

**Figure 4:**
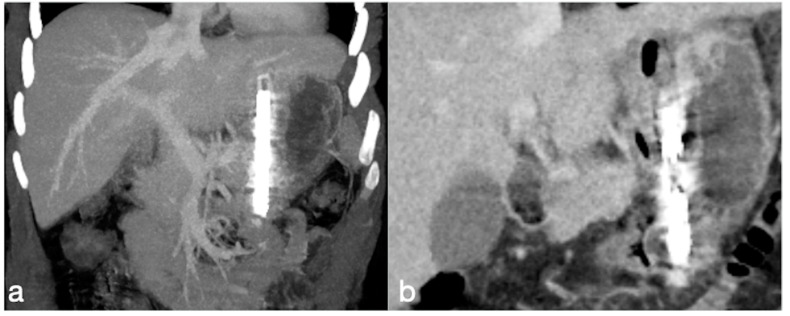
A correct positioning of BariClip^®^ seen at CT coronal MIP (**a**) and MPR (**b**) views.

CP procedure reproduces the effect of the sleeve gastrectomy with the advantage of the reversibility by clipping and not cutting the stomach. By using a laparoscopic approach, the BariClip^®^ is placed into the peritoneal cavity. The BariClip is then closed around the stomach parallel to the lesser curvature, creating a small medial pouch and an excluding large lateral segment (Figure 4). To prevent slippage, the BariClip^®^ is sutured to the gastric wall both anteriorly and posteriorly at various levels of the stomach [15].

### 2.2. Combined Restrictive and Malabsorptive Procedures

Combined restrictive and malabsorptive procedures include Roux-en-Y gastric bypass (RYGB) and one anastomosis/mini-gastric bypass (OAGB-MGB). 

**Figure 5:**
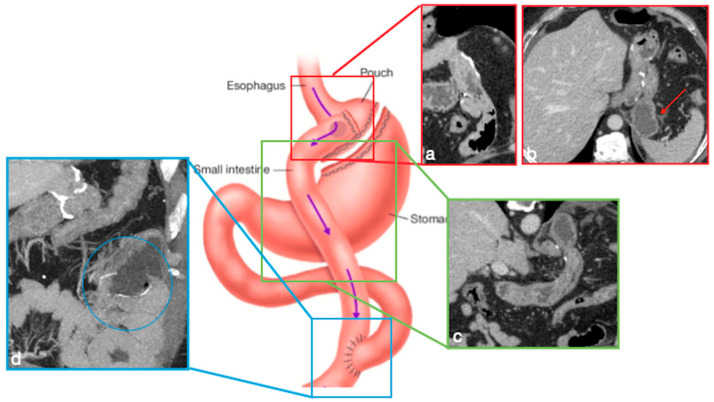
Gastric bypass. Surgical sutures seen at CT. Gastric pouch with gastrojejunostomy (**a**,**b** axial oblique view) and excluded stomach (**b**, arrow). Roux limb (**c**, coronal oblique view) of length of 75 to 150 cm from the jejunal division point for an average of 120 cm, jejunojenuostomy (**d**, circle, coronal view) between the biliopancreatic limb and the distal segment of jejunum (roux limb). The scheme was adapted from https://www.uptodate.com/contents/image/print?imageKey=GAST%2F79256 (accessed on 23 October 2022).

In RYGB, the transit of food along the gastrointestinal tract is rerouted to achieve a reduction in the absorptive capacity for calories. A small gastric pouch results from a surgical exclusion of the remnant stomach, followed by a gastrojejunostomy (between the new gastric pouch and the jejunum: alimentary limb) and a jejunojejunostomy (between the duodeno–jejunum channel and the jejunum–biliopancreatic limb), placed in communication in a common channel further downstream (Figure 5) [4,6].

**Figure 6:**
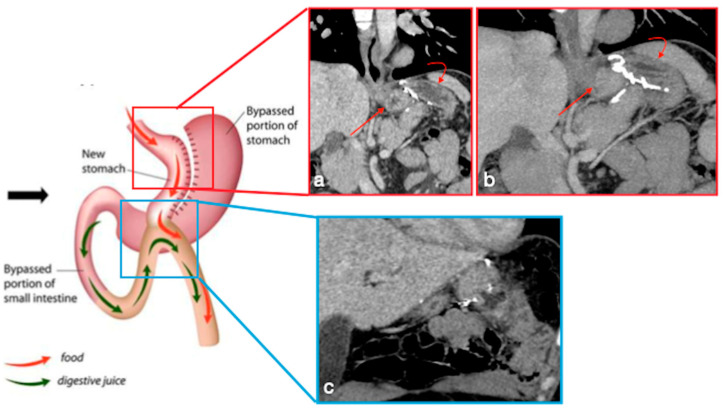
Mini-gastric bypass. Surgical sutures seen at CT. Gastric pouch (**a**,**b**, coronal view (**a**) and MIP (**b**) straight arrows). Bypassed stomach (**a**,**b**, curved arrows). Gastrojejunostomy (**c**, coronal oblique view). The scheme was adapted from https://www.theossi.com/mini-gastric-bypass-obesity-surgery.html (accessed on 23 October 2022).

In OAGB-MGB an endo-stapler (from the His angle to 2 cm proximal to the pylorus) is used to create a mini-gastric tube, which is connected with the jejunum via a side-to-side antecolically anastomosis, in order to bypass gastric antrum, duodenum, and proximal jejunum (Figure 6) [16]. The goal of the gastric pouch is to remove the reservoir function of the stomach and convert the stomach into a nonobstructed extension of the esophagus, where food does not stay in a reservoir but is dumped into the lumen of the jejunum. The presence of gastrojejunostomy causes malabsorption [16,17].

## 3. Complications

### 3.1. Imaging Techniques

Patients with suspected complications may be imaged with:

- Upper gastrointestinal (UGI) barium studies;
- Fluoroscopic upper GI examinations with water-soluble contrast agents; and
- Enhanced-CT.

Upper GI barium studies are mainly used for detecting stomal stenosis, dysfunction of the jejunojejunostomy, band slippage, and for assessing routine band adjustments, whereas fluoroscopic upper GI examinations with water-soluble contrast agents find application in suspected leaks when CT is not available, both of them in an elective setting.

Enhanced CT represents the gold standard imaging examination in an emergency setting.

The CT imaging protocol in an emergency setting is necessarily wide and should include an unenhanced scan, unless getting the virtual unenhanced scans from dual energy acquisitions, followed by contrast-enhanced scans (a 400-mg iodine/mL contrast medium is suggested with a variable volume of 130–150 mL at an injection rate of 3–5 mL/s) acquired in the arterial phase (bolus tracking), portal venous phase images (70-s delay) and, eventually, in the late phase (about 180-s delay). The administration of hydrosoluble contrast medium may be helpful only in selected cases; indeed, in several cases signs of leaks are already detectable without administration of endoluminal contrast medium (e.g., suture dehiscence, fistulous tract, extraluminal air bubble, air-fluid collections). Furthermore, the adoption of thin slices and reconstruction indices (1–2.5 mm) to obtain good MIP (maximum intensity projection) and MPR (multiplanar reconstruction) is helpful for diagnosis [18].

### 3.2. General Complications, Procedure Unrelated

The comorbidities of people with obesity (e.g., diabetes mellitus, cardiovascular and respiratory disorders) predispose these patients to higher risk of complications when going under general anaesthesia and having surgery [1,12].

Complications may occur intraoperatively or postoperatively.

Intraoperative complications include anaesthesia-related events, injury to bowels, spleen, liver, or damage to a major blood vessel, bowel ischemia, trocars insertion, technical errors, and difficult or aberrant anatomy. The main postoperative complications in the bariatric surgery are the same as in any surgery: myocardial infarction, deep vein thrombosis, pulmonary embolism, haemorrhages, and infections [12,19,20,21,22].

### 3.3. Specific Complications

#### 3.3.1. GB

GB placement is associated with a complication rate reported up to 5.5% [23] Significant complications include spontaneous deflation and migration of the balloon, peptic ulcers, gastric outlet obstruction, and gastric perforation.

- Spontaneous deflation-rupture

**Figure 7:**
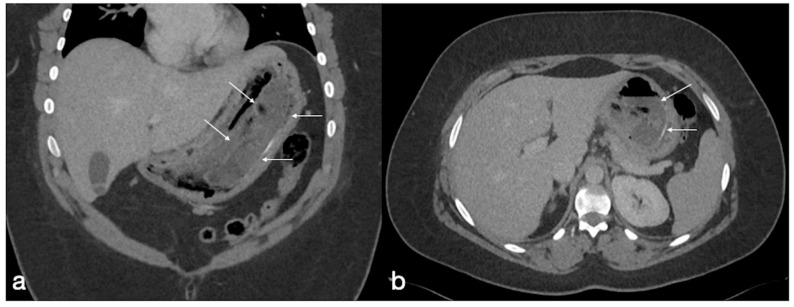
Deflated gastric balloon. A 17-year-old female patient with gastric balloon placed two months before, came to the emergency department complaining of a change in urine color due to spreading of methylene blue from the balloon lumen. A CT was requested to evaluate balloon position. The deflated balloon is still in the gastric cavity (**a**, coronal oblique view, **b** axial view, arrows).

GB complication may include spontaneous deflation in about 6% of cases [23]. Balloons are inflated with blue-dyed saline, and patients generally note grey or blue urine, derived from spontaneous deflation followed by gastrointestinal absorption of blue dye (Figure 7) [10].

Imaging findings:

Deflated gastric balloon into the gastric cavity (Figure 7).

- Ulceration

A balloon standing in the stomach more than six months represents a condition with intrinsic risk of perforation, as a result of long-term pressure, wall necrosis, and ulceration, as well as gastric outlet obstruction [10]. The suspect is guided by the clinical history of prior surgery and patient’s symptoms are often nonspecific [24].

Imaging findings:

In case of perforation, the UGI may reveal signs of intraabdominal free air and a CT can detect the balloon within the stomach with intraperitoneal free air [25,26].

- Gastric outlet obstruction

Gastric outlet obstruction rarely occurs; indeed, it is reported that about 0.76% of balloons are complicated by gastric obstruction within the first two weeks of placement [10,23]. An early recognition and intervention is required, as gastric outlet obstruction secondary to an intragastric balloon may progress to necrosis and perforation.

Imaging findings:

UGI may show signs of gastric overdistension associated with impaired progression of endoluminal contrast medium.

**Figure 8:**
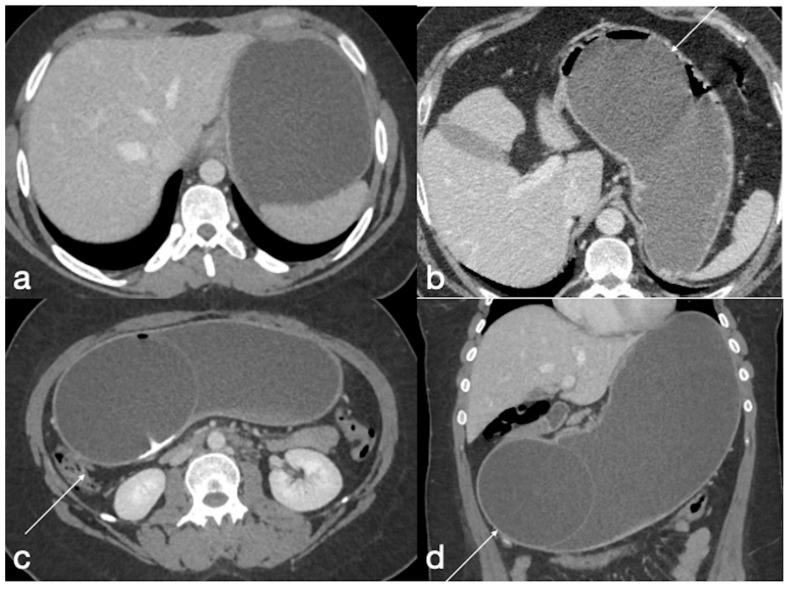
Gastric outlet obstruction. A 28-year-old female patient with a gastric balloon came to the emergency department complaining of sudden abdominal pain followed by persistent vomiting. Note the stomach overdistension proximal to the balloon, displaced in the antro-pyloric region (**a**–**c**, axial views, **d**, coronal view; balloon, arrows).

The CT shows in detail the balloon within the gastric antrum associated with variable distension of the gastric lumen (Figure 8) [23].

- Migration and bowel obstruction

Deflated/ruptured balloon can migrate into the small bowel, causing small bowel obstruction.

**Figure 9:**
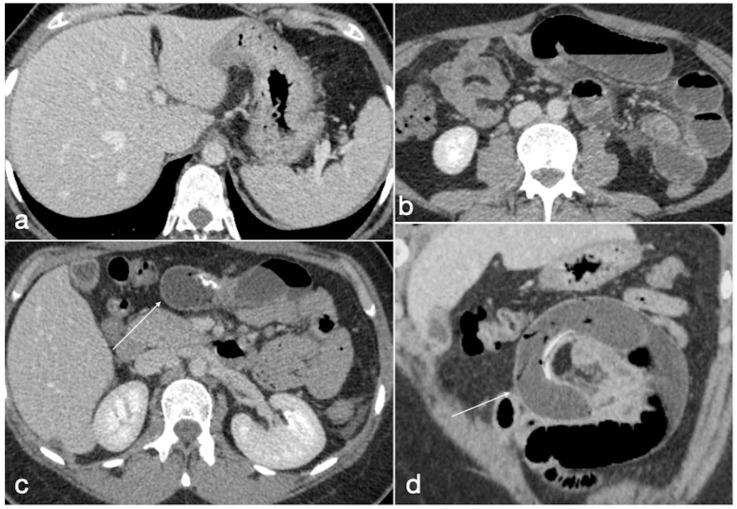
Migration of ruptured balloon with SBO. A 30-year-old female patient with a gastric balloon came to the emergency department complaining of abdominal pain and constipation. The balloon is displaced as the gastric cavity is empty (**a**, axial view). There are signs of mechanic ileus with air-fluid mixed stasis in the small bowel (**b**, axial view) proximal to the ruptured balloon (**c**, axial view, arrow) causing SBO, best seen in the coronal view (**d**, arrow).

The migration of endogastric balloons [10] rarely occurs (0.3–1.4% of cases [27]) and is related with balloon rupture and deflation followed by migration in the small bowel causing small bowel obstruction, which may occur in 0.7–0.8% of cases (Figure 9) [10]. Even in the deflated state, the migrated balloons are large, so an enterotomy is usually necessary to remove the balloon.

Imaging findings:

Signs of bowel obstruction are detectable on both abdominal X-ray and CT.

The CT is able to identify the detailed position of the deflated balloon, seen as a tubular foreign body, with a proximal small bowel dilatation (Figure 9). A CT (with intravenous contrast medium) is necessary to evaluate the severity of the bowel obstruction and the presence of further complications (e.g., GI perforation), and to guide surgery.

#### 3.3.2. AGB

- Band slippage

The incidence of band slippage is reported to range between 4 and 13% [6]. It consists of a band dislocation with consequent herniation of the stomach above the band, with resultant eccentric pouch dilatation. The slippage could be anterior, posterior, or concentric (when a complete distal dislocation of the band is observed) [4].

**Figure 10:**
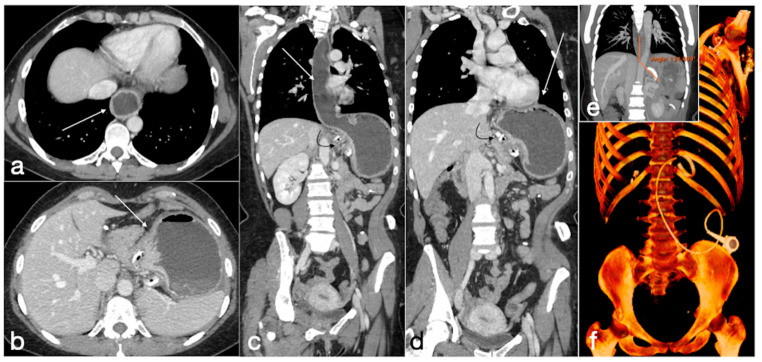
Gastric band slippage with gastric obstruction. A 40-year-old female with known gastric banding was complaining of abdominal pain and vomiting. There is dilatation of the esophagus (**a**, axial view, arrow) and of the gastric lumen proximal to the gastric banding (**b**, axial view, arrow). These findings are best seen in the coronal views (**c**,**d**, esophagus and stomach, straight arrows; gastric banding curved arrows). In (**e**) is shown the phi angle, excessively wide (134,699°), and in (**f**) the volume rendering reconstruction in which the excessive angulation of the device is clearly seen.

Imaging findings:

On an AP radiograph, the superior angle between the longitudinal axis of the gastric band and the spinal column (φ angle, phi) should be from 4 to 58 degrees, and the correct spatial position of the gastric band is approximately 5 cm below the left hemidiaphragm. The distal displacement of the band, with an increased distance between the band and the left hemidiaphragm [28] and a change in band orientation (with rotation along the horizontal axis and loss of overlapping between the anterior and posterior portions of band that results in “O” shape visible on AP projection [4]) is associated with value alterations of the superior angle composed of the longitudinal axis of the gastric band and the spinal column (φ angle), as measured by AP projection (Figure 2 and Figure 10) [28].

- Pouch dilatation

A pouch dilatation with normal or enlarged stoma usually occurs in the case of dietary noncompliance and chronic pouch overfilling (reported in 3–8% of cases). 

Imaging findings:

Imaging (UGI or CT) shows a dilatation of proximal pouch [3] that should measure 4 cm in diameter (15–20 mL) [28] generally, with a concentric appearance and patent stoma. When a narrowed stoma is present, the morphology of the enlarged pouch is very important because a concentric aspect is indicative of overinflation of balloon cuff, and eccentric appearance is caused by band slippage [4].

CT examination is the best imaging method by which to perform these evaluations, allowing measurement of the pouch and also of the stoma diameter, which is considered normal at less than 4 cm [4,28].

- Device-related

The incidence of device-related complications is reported between 1.4 and 26%, depending on follow-up timing, and often requires surgical repair. Infection of port, tube, or band is the first cause, occurring in 6% of cases; the reservoir migration in soft tissue with the possibility of becoming inverted occurs in 3% of cases. Another possible complication is represented by an altered integrity of port components [4]. 

**Figure 11:**
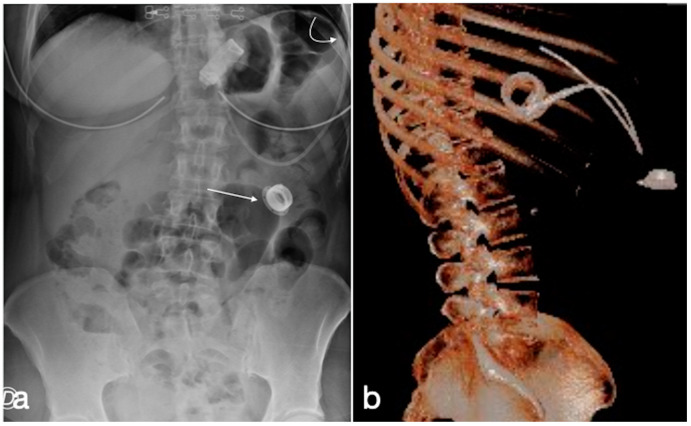
Gastric banding, detachment of the catheter from the port. A female patient in whom it was no longer possible to adjust the gastric banding. In the CT scout, the detachment of the catheter extremity (**a**, curved arrow) from the port (**a**, straight arrow) is clearly evident. In (**b**), the same findings are shown in the volume three-dimensional reconstruction.

Imaging findings:

Both X-ray and CT may detect migration or rupture of the device; however, CT allows one to study in detail the port components (Figure 11) and also to examine soft tissues alterations adjacent to the port. This is important, for example, in case of fluid collection due to soft tissue infection.

- Erosion

The band can erode the gastric wall partially or completely with displacement into the lumen or migration [29]. The incidence of gastric wall erosion is close to 2%, and this complication is more frequently detected in a longer follow-up, resulting from persistent decubitus of the gastric band with high pressure on the gastric wall that may cause necrosis and wall erosion [6].

Imaging findings:

On UGI, the contrast will surround the intraluminal portion of the band, resulting in an intraluminal filling defect and contrast may will not cross over the stoma.

At CT, erosion will be visible when the band is completely intraluminal or when the contrast medium distributes around the intraluminal portion. Therefore, CT accurately detects associated abscess or peritonitis [4].

- Acute perforation

Gastric perforation during a laparoscopic banding is an uncommon complication, occurring in less than 1% of cases, and which may develop after a traumatic event involving the gastric wall.

Imaging findings:

The UGI could show a contained or free extravasation of water-soluble contrast material around the perforated point. A CT reveals extraluminal gas or fluid collection in the left upper quadrant [12].

- Gastric volvulus

In very rare cases of band slippage, a twisting of the prolapsed proximal stomach around the band may occur, causing a closed-loop obstruction. Surgery is needed to remove the band in order to avoid a gastric infarction or perforation.

Imaging findings:

The UGI with an oral contrast medium reveals the proximal stomach dislocation around the band and the gastric body rotation upward and to the left. The CT, with intravenous contrast medium, is useful to detect gastric ischemia or infarction with gastric wall thickening and pneumatosis [30].

#### 3.3.3. SG

- Leak

This is historically known as the primary complication post-SG [4], generally occurring in the first 30 days. However, recent studies report an incidence equal to or lower than 0.15% in selected low-risk patients [31]. The most common localization is the proximal region of the staple line, near the gastroesophageal junction, related to the relatively poor perfusion in this location. Common symptoms are tachycardia, abdominal pain, and fever.

Imaging findings:

A UGI examination with water-soluble contrast may show extraluminal tracks or collections in the left upper quadrant [4,6].

**Figure 12:**
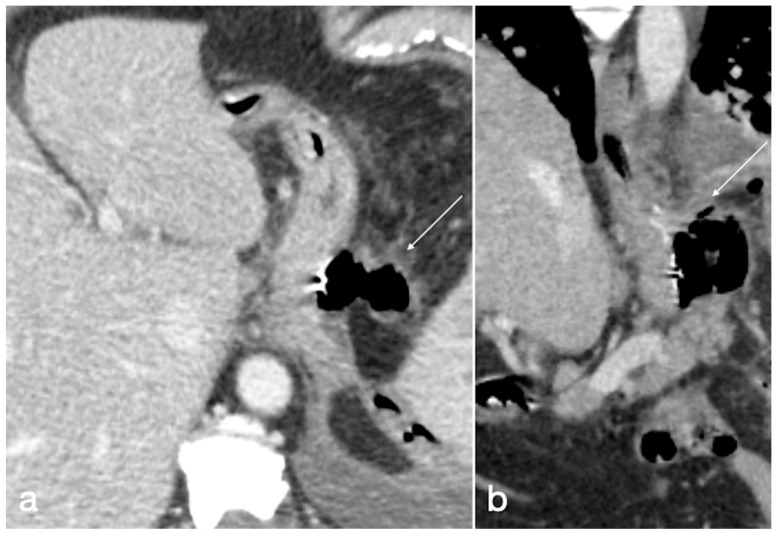
Type I leak after sleeve gastrectomy. See the air collection adjacent to the surgical suture (**a**, axial view, arrow; **b**, coronal view, arrow).

**Figure 13:**
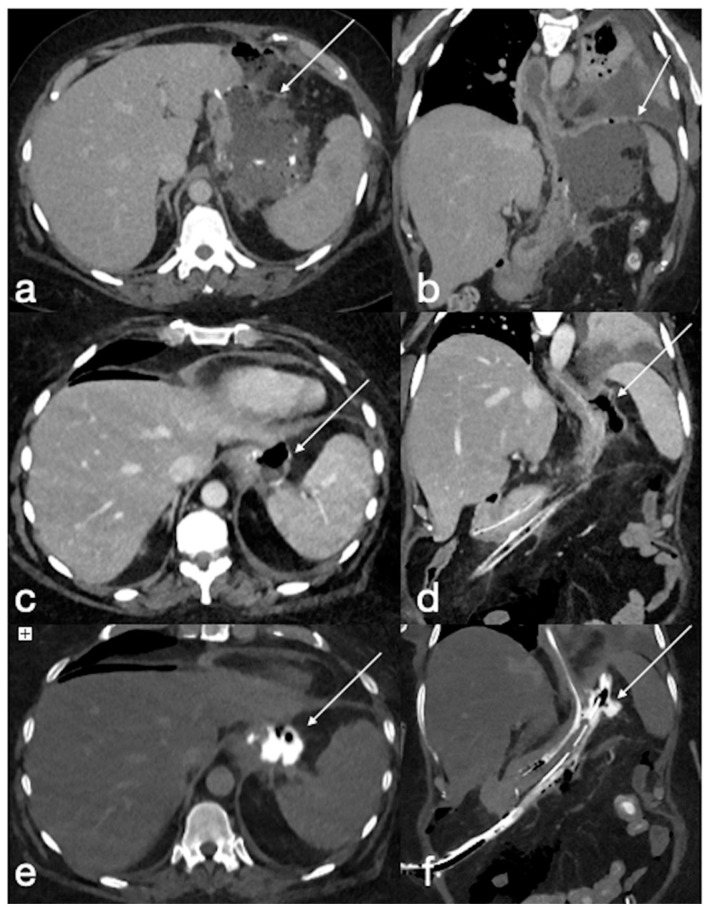
Type III leak after sleeve gastrectomy (**a**, axial, **b**, coronal oblique view, arrows) requiring surgical re-suturing. Eight days later, the CT again showed an air–fluid collection adjacent to the proximal gastric suture (**c**, axial, **d**, coronal oblique view, arrows) with extraluminal spreading of oral contrast medium (**e**, axial, **f** coronal oblique view, arrows). After any attempt at conservative treatment, the patient underwent total gastrectomy.

The CT may evidence the same findings, but with higher sensitivity. Furthermore, CT add more information, such as the presence of fluid/blood collection or abscesses (Figure 12 and Figure 13). A small proximal gastric pouch created to preserve a part of fundus at surgery could be a leak mimicker at UGI [4].

1. After SG leakage classification

According to the most recent leakage classification from Montpellier University Hospital, the first step is the characterisation of the collection type at CT.

Type I is a collection <5 cm in the left upper quadrant (LUQ), with a leak that could be not visible on CT (a, no leak) or visible (b, positive leak). 

Type II is found when the collection is >5 cm in LUQ. Moreover, in this case it is classified as a (no leak) or b (positive leak). 

Type III is for diffuse abdominal collections.

Type IV is found in cases of pleural (thoracic) collections.

Therefore, based on the collection localization to the staple line, it is possible to describe a leak in the superior (S), middle (M), or inferior (I) part of the sleeve.

Generally, for the types II, III, and IV, surgical drainage is necessary (Figure 13), whereas type I is clinically well-tolerated, and intervention can be avoided [32].

- Gastric dilatation

In subjects with no evidence of adequate weight loss or recurrent weight gain, UGI imaging studies can reveal a widening of the new stomach with no more tubular appearance. This complication is estimated to occur with an incidence of 4.5% [6].

- Stenosis

**Figure 14:**
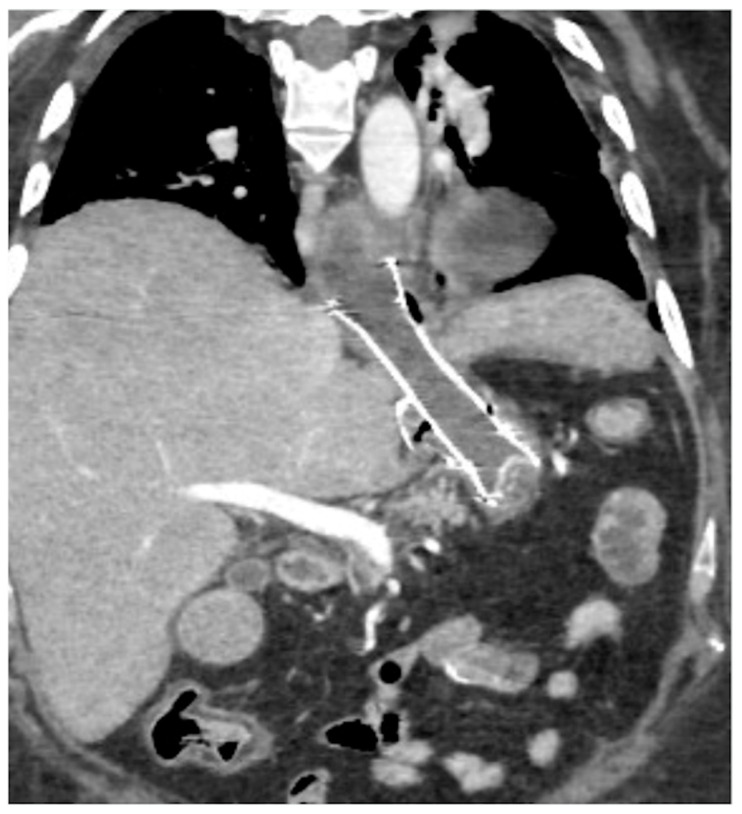
Stenting of proximal stricture of the gastric suture. Coronal oblique view.

This complication is reported in about 3.9% of patients who develop gastric obstruction symptoms and marked narrowing of the pouch, resulted from edema or ischemia (early post-operative time) or staple line scarring and fibrosis (late post-operative time). Strictures usually occur in the most narrow portion of the sleeve (at the site of incisura angularis) and can be treated endoscopically with dilation and stenting (Figure 14), but in serious cases, they require surgical revision or conversion to RYGB.

Imaging findings:

UGI imaging studies may reveal focal strictures or segmental narrowing with delayed or absent transit of endoluminal contrast medium. The CT, with the eventual administration of oral contrast medium, offers a better definition of the stenotic area [33].

- Spleno-porto-mesenteric vein thrombosis (PVT)

The described incidence is around 0.3%. PVT is difficult to recognize, and symptoms are vague and not specific, but consequences are significant. This complication can occur in any of the other mentioned obesity laparoscopic treatments, but the incidence after SG is higher, probably due to the ligation of gastroepiploic vessels or short gastric vessels. During this procedure, performed in close proximity to the splenic vein, it may occur as a mechanical injury to the vein as a possible cause of thrombosis; another possible cause of thrombosis may be the alteration of venous return from the stomach. It may also occur as a consequence of post-discharge dehydration and hypovolemia [7].

**Figure 15:**
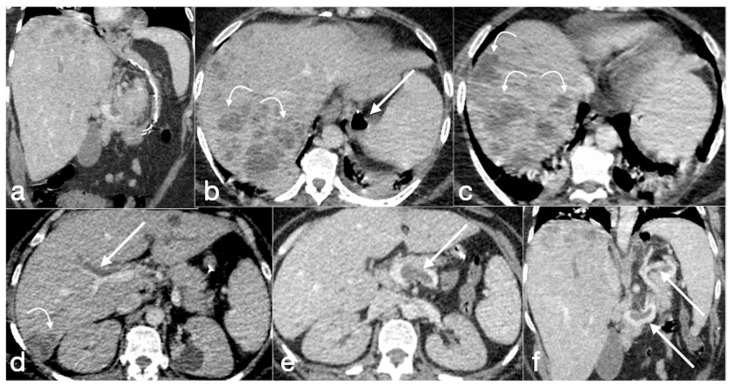
Spleno-portal thrombosis and liver abscesses after sleeve gastrectomy with suture leak. A 46-year-old female with a type I leak after sleeve gastrectomy complained of abdominal pain, fever and kidney failure. Note the gastric surgical suture (**a**, coronal oblique view), the presence of a type I leak (**b**, axial view, straight arrow), and of multiple liver abscesses (**b**–**d**, axial view, curved arrows) related to extensive spleno-portal thrombosis (**d**–**f**, straight arrows). The leak is responsible for spread of gastric content and bacteria, causing septic spleno-portal thrombosis and, consequently, liver abscesses. The leak was conservatively treated and the thrombosis was successfully treated with multiple sessions of transhepatic catheter-directed thrombolysis.

Imaging findings:

Color Doppler Ultrasound may detect flow defects in explorable vessels; however, CT has major panoramicity and is suggested as the first imaging examination in these patients (Figure 15) as it can depict in detail the extension and severity of the thrombotic defects [7].

#### 3.3.4. RYGB

- Stenosis

Stenosis has been reported in 3–9% of patients with obstructive symptoms. In the early postoperative period stenosis could be due to hematoma or edema at gastrojejuno or jejunojejuno anastomosis, whereas in the late postoperative period fibrosis caused by postsurgical scarring or chronic ischemia resulted from tension on the gastrojejuno anastomosis are the main causes (tension on the jejunojejuno anastomosis is very uncommon) [34].

Imaging findings:

UGI examination will show a segment of smooth narrowing in correspondence of gastrojejuno-anastomotic stricture, with dilatation of the gastric pouch if stricture causes obstruction and delayed transit of intraluminal medium contrast. Anastomotic strictures in inferior position in relation to the gastric pouch are well-detected in frontal projection, but strictures in anastomosis that have anterior or posterior location cannot be readily documented in frontal projection for the overlap between gastric pouch and alimentary limb. A steep oblique or lateral view scan should be performed to eliminate overlaps. 

Patients generally have a good response to endoscopic dilatation of the strictures [6]. The CT allows us to better characterise the stenotic tract and to detect further complications eventually associated.

- Leak

Extraluminal leaks represent an important complication of RYGB, most often occurring within 10 days after surgery; in selected low-risk patients, this incidence is reported to be lower than 1.3% [31]. The gastrojejunal anastomosis is involved in 69–77% of cases, but other localizations are possible, such as gastric pouch, blind-ending jejunal stump, and jejunojejunal anastomosis [6].

Imaging findings:

Early diagnosis is essential because the risk of abscess formation, peritonitis, and sepsis.

Leaks after RYGB generally have extension into the left upper quadrant, to the left gastrojejunal anastomosis and loculated or free extraluminal gas could be present. UGI examination after administration of water-soluble contrast allows us to visualize leaks as blind-ending tracks or collections from the gastrojejuno anastomosis with the patient in a supine or supine left posterior oblique position. At CT, it is also possible to recognize subtle leaks and to easily differentiate a breakdown of gastric staple line with the development of gastrogastric fistula [4,6].

- Small bowel obstruction

**Figure 16:**
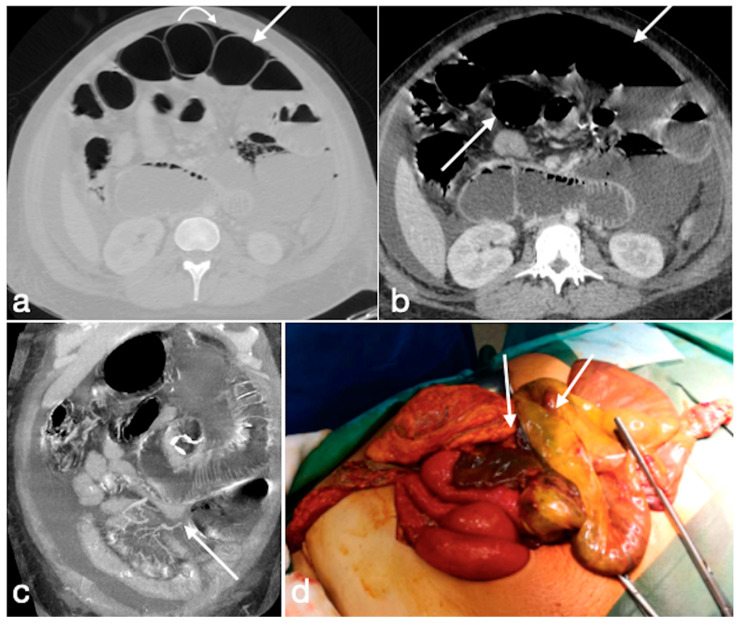
Internal hernia with bowel necrosis after cesarean delivery. A 37-year-old-female patient complaining of abdominal pain, fever, and constipation early after cesarean delivery. Note the presence of intraperitoneal free air (**a**, axial view, curved arrow), overdistension of a small bowel loop with thin wall, some of them with barely noticeable enhancement (**a**,**b**, straight arrows), and the convergence of bowel, mesentery, and vessels of the closed loop at the hernia orifice (**c**, coronal view, straight arrow). The prompt volume reduction of the lower abdomen is probably due to the delivery, which solicited the bowel herniation. The patient underwent prompt surgery confirming the CT diagnosis and leading to bowel resection due to necrosis (**d**, straight arrows, bowel loops in different stages of ischemia/infarction). Image courtesy of Dr. Michele Lanza, Dr. Antonio Brillantino and Dr. Maurizio Castriconi Department of Emergency Surgery, “A. Cardarelli” Hospital, Naples, Italy.

**Figure 17:**
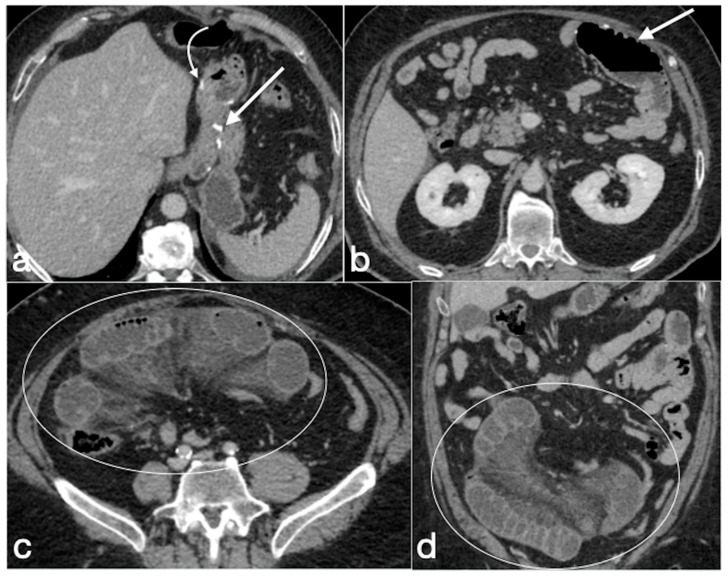
Small bowel volvulus in patient with gastric bypass. A 55-year-old male patient was complaining of acute abdominal pain and constipation. See the gastric bypass (**a**,**b** axial oblique view; **a**, straight arrow, gastric suture; **a**, curved arrow, gastrojejunostomy; **b**, straight arrow jejunojejunostomy) and the closed loop obstruction due to volvulus (**c**, axial view, circle; **d**, coronal view, circle).

The main causes of this complication (incidence rate 5%) are internal hernias (Figure 16), adhesions (Figure 17), hernias of the anterior abdominal wall, anastomotic strictures and, less frequently, intussusceptions [35,36,37,38]. 

More rarely, small bowel obstruction occurs within the 30-day postoperative period (incidence: 1.7%) [35], is defined as “early small bowel obstruction” (ESBO) [35], is mostly located at the jejunojejunostomy (JJ), and is procedure-related (mesenteric defects closed with internal hernia, alimentary limb mesenteric ischemia, jejunojejunostomy narrowing, adhesions, twisted alimentary limb). 

Clinical presentation of small bowel obstruction can be limited to nausea and vomiting, and the prevalence of this complication in patients who received antecolic position of alimentary limb is not clear [36,38].

Imaging findings:

There are three different patterns of SBO, detectable with both UGI and CT examinations.

(a) The obstruction involves the alimentary limb, that appears dilated, while both biliopancreatic limb and distal common channel are hypotonic.
(b) The obstruction involves the biliopancreatic limb, which appears dilated, whereas the alimentary limb and distal common channel are decompressed. This closed-loop obstruction could determine perforation of excluded gastric cavity. On UGI, there is a dilated and fluid-filled limb (biliopancreatic) excluded by an oral contrast passage that could determine a mass-effect on the other bowel loops. Upon CT, detection is easy, and it should be suspected not only for the biliopancreatic limb appearance associated to decompression of alimentary and common channel, but also for the recognition of dilatation of excluded gastric room.
(c) The obstruction involves the common channel; any bowel loops upstream appear dilated [4].

- Fistula

There is a pathologic communication between the newly generated gastric pouch and the native gastric cavity, generally due to incomplete surgical transection. The first manifestations are weight gain or minimal weight loss, pain, and ulcers [4].

Imaging findings:

The gold standards for diagnosis are reported to be endoscopy and the barium contrast study, which easily reveals a subtle tract of communication between the two gastric rooms. In reality, CT better depicts that this complication is also valid. The treatment of resection and re-suture is laparoscopy-based [39].

- Internal hernia

Generally considered a late complication, the internal hernia has a reported incidence of 1–5% and may occur at any time after surgery (Figure 16) [40]. The incidence rates depend largely on the surgical technique. The herniation into mesenteric defect can determine bowel obstruction, ischemia, infarction, and perforation. There are three characteristic mesenteric defects, typically created in the transverse mesocolon for a retrocolic Roux limb, near the jejunojejunal anastomosis and posteriorly to the Roux limb (Petersen’s defect). 

Imaging findings:

Symptoms could be nonspecific and UGI or CT findings are often difficult to interpret in postsurgical configuration and anatomy. Signs that led to diagnosis are. the migration of the suture line, visible on both UGI and CT. Note also that the herniated loop displaces the other bowel loops, frequently into the left abdomen (90%). Stasis of contrast media in the loops could be detected as a sign of obstruction or an obstacle to the physiologic intestinal canalization; swirling and stretching of mesenteric vessels with mesenteric edema and engorged mesenteric nodes could be depicted by CT as well as the tubular or round shape of peripheral mesenteric fat closely surrounded by bowel loops (‘‘hurricane eye sign’’) [4,19,40,41,42,43,44].

- Marginal ulcers

Gastrojejunal ulcers are generated by chronic exposure of the mucosa to acid arriving in the Roux limb, and patients typically present epigastric pain.

Imaging findings:

Even if the UGI barium study is currently less used, it allow us to depict single ulcers, varying in size, at the gastrojejuno anastomosis or in the Roux limb adjacent to the anastomosis. Endoscopy represents the diagnostic method of choice. Treatment is medical, and occasionally surgical for untreatable ones.

- Intussusception

This complication is rare. In the literature, two cases of adults with intussusception after RYGB are described; they showed signs and symptoms of small bowel obstruction [45,46].

#### 3.3.5. OAGB-MGB

- Marginal ulcers

Reported ulcer incidence after OAGB-MGB vary between 0.5% and 5.0% [47,48,49]. Comparing OAGB-MGB and RYGB data, no significant differences are found in incidence rate and imaging findings [50].

- Internal hernia

An incidence of 3.9% of internal hernia after OAGB-MGB [50,51,52] is reported. Imaging findings are similar to internal hernia after RYGB, already described.

- Anastomotic stenosis

Anastomotic stenosis is considered an early complication (from the third day to one month after the operation) either due to postsurgical edema and strictures or technical errors, such as anastomotic breach, suture failure, intestinal limb malpositioning, or stapler misfiring [52,53].

However, a relatively recent multi-institutional survey on 2678 patients with a mid-term (5 years) follow-up showed that the anastomotic stenosis may occur also as a late complication (from month 2 up to the 10 years) [54] related to adhesion subsequent to bariatric surgery [55].

**Figure 18:**
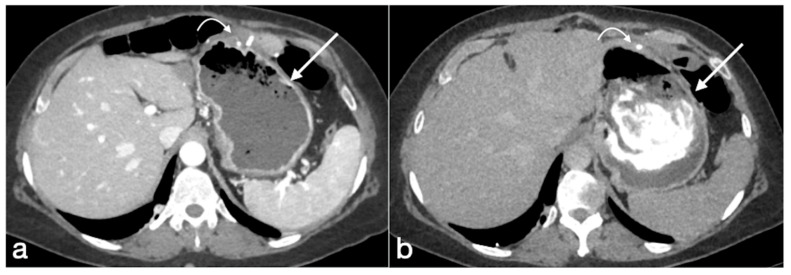
Relapse of anastomotic stricture in patients with mini-gastric bypass. A 58-year-old patient underwent previous gastric bypass followed by surgery for anastomotic stenosis, and came back still complaining of abdominal pain and dyspepsia. See the dilated stomach (**a**,**b** axial views, straight arrows), proximal to the anastomosis (**a**,**b**, curved arrows) with endoluminal stasis of the iodinated contrast agent that was orally administered (**b**, straight arrow).

The incidence of anastomotic stenosis after OAGB-MGB reaches 0.2–0.4% [54] (Figure 18).

Upon CT, after contrast media administration, it is possible to demonstrate the distention of the excluded gastric lumen caused by the narrowing at the gastrojejunal anastomosis [53].

The reported management may consist in laparoscopic repair, endoscopic balloon, or conservative treatment [54].

### 3.4. Unexpected Complication

**Figure 19:**
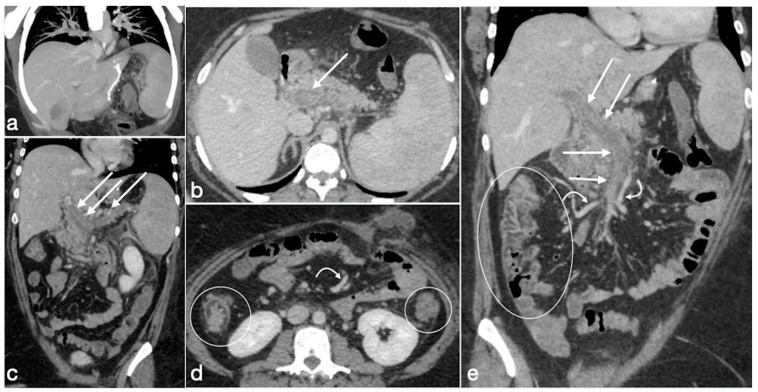
Patient 1. Porto-spleno-mesenteric venous thrombosis and colonic ischemia after sleeve gastrectomy. A 37-year-old female patient underwent sleeve gastrectomy. The patient came to the emergency department complaining of abdominal pain and fever. See the surgical gastric suture (**a**, coronal oblique view), the enlarged and thrombosed spleno-portal lumen (**b**, axial view; **c**, coronal oblique view, straight arrows), the consequential mesenteric congestion (**d**, axial view; **e**, coronal view, curved arrows), and the colonic ischemia (**d**, axial view; **e**, coronal view, circles). In the coronal view (**e**), the thrombosis that extends to the superior mesenteric vein lumen can be seen best. The patient was treated with multiple session of transhepatic catheter-directed thrombolysis.

**Figure 20:**
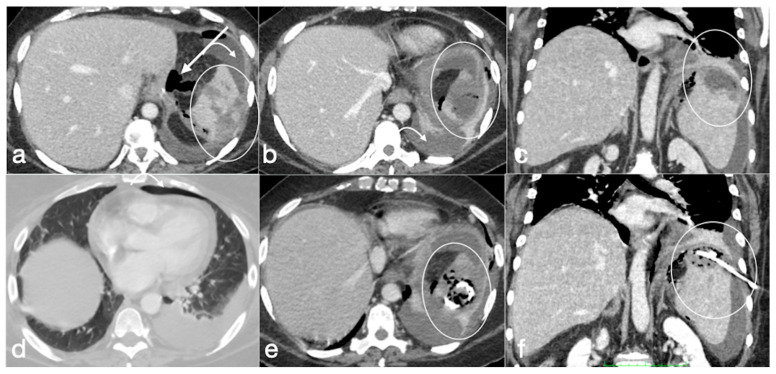
Patient 2. Suture leak with spleen abscess after sleeve gastrectomy. A 48-year-old patient underwent sleeve gastrectomy and complained in the following days of abdominal pain, fever, and dyspnea. At the first CT examination is seen the leak (**a**, axial view, straight arrow), free peritoneal fluid (**a**, curved arrow) and inhomogeneous enhancement of the spleen (**a**, circle). In more cranial scans, a splenic abscess is seen (**b**, circle) associated with the reactive pleural effusion (**b**, curved arrow), and the coronal view (**c**) further clarifies the finding (**c**, circle, splenic abscess). It was decided that the patient should be managed with percutaneous drainage of the splenic abscess. The patient came back after the drainage positioning, with chest pain and dyspnea (**d**–**f**) and at CT the left anterior pneumothorax (**d**, axial view, curved arrow) was detected due to the drainage positioning, which is actually in the splenic abscess (**e**, axial view, circle). However, in its course it crosses the left diaphragm (**f**, coronal view, circle).

**Figure 21:**
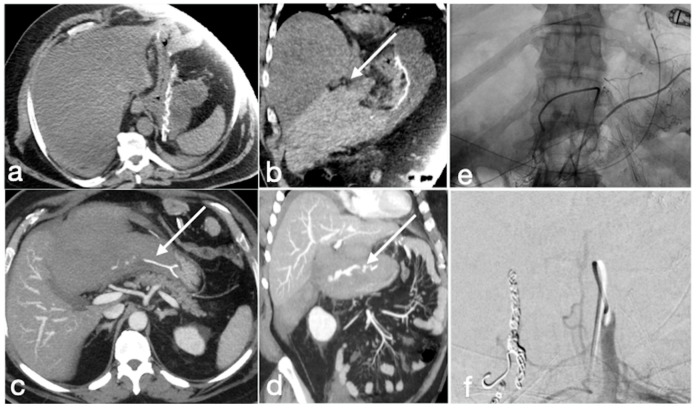
Patient 3. Huge hematoma with active bleeding. A 48-year-old patient developed abdominal pain and tachicardia suddenly after surgery for mini-gastric bypass. See the surgical suture (**a**, axial view). There is also a large hyperdense collection (**b**, coronal view, arrow) with active arterial bleeding (**c**, axial view, arrow), increasing conspicuously in the following portal-venous phase (**d**, MIP coronal view, straight arrow), arising from the gastroduodenal artery (branch of hepatic artery). It was promptly treated by gastroduodenal artery embolization (**e**,**f**).

**Figure 22:**
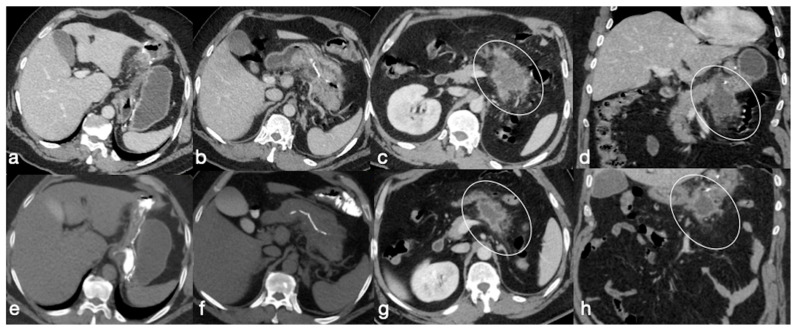
Patient 4. Leak from excluded stomach after gastric bypass. A 47-year-old male patient with previous gastric bypass was complaining of abdominal pain and fever. Note the surgical sutures of the gastric bypass (**a**,**b**, axial view) and in (**c**) the fluid collection (circle) that seems to arise from the excluded stomach (**d**, circle) and not supplied after the oral contrast administration (**e**,**f** axial views). According to the interventional radiologist and the surgeon, it was decided to conservatively treat the patients and at the CT follow-up 13 days later the fluid collection was reduced (**g**, axial view; **h**, coronal view, arrows).

**Figure 23:**
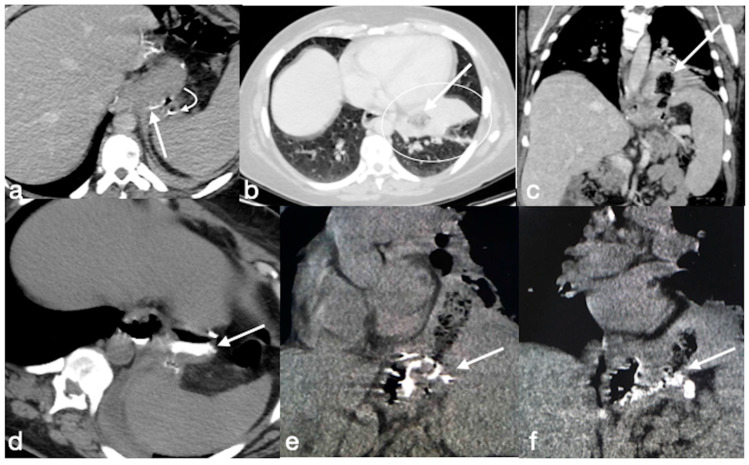
Patient 5. Gastro-bronchial fistula after sleeve gastrectomy. A 52-year-old female was complaining for persistent cough and fever more than one month after sleeve gastrectomy followed by a leak conservatively treated. See the surgical suture of the sleeve gastrectomy (**a**, axial view, straight arrow), close to which there is a small hyperdensity (**a**, curved arrow). Furthermore, there is a pulmonary consolidation (**b**, axial view, circle) in the context of which there is an inhomogeneously hypodense round collection with some air bubbles suspected for pulmonary abscess (**b**, arrow). This area appears continuously to the gastric suture, and it is best seen in the coronal view (**c**, arrow). After the hydrosoluble oral contrast administration, this migrates into the abscess (**d**, axial view; **e**,**f**, coronal view; arrows) that was demonstrated to be related to the presence of a gauze.

Several cases of unexpected complications are here reported [Figure 19, Figure 20, Figure 21, Figure 22 and Figure 23].

## 4. Conclusions

In conclusion, several complications, more or less expected, may be encountered in patients who underwent bariatric surgery. The diagnostic imaging method of choice is represented by an enhanced CT that allows us to obtain a complete assessment of the patient condition and to plan an adequate treatment. To correctly interpret the imaging finding and to perform a correct and detailed diagnosis, radiologists should know the basis of the surgical technique and of the postoperative anatomy.
